# Novel recombinant R-spondin1 promotes hair regeneration by targeting the Wnt/β-catenin signaling pathway: Recombinant R-spondin1 promotes hair regeneration

**DOI:** 10.3724/abbs.2023112

**Published:** 2023-07-20

**Authors:** Yijun Chen, Zhujin Lu, Jiaxin Feng, Zefeng Chen, Zejian Liu, Xiuqi Wang, Huichao Yan, Chunqi Gao

**Affiliations:** College of Animal Science South China Agricultural University/Guangdong Provincial Key Laboratory of Animal Nutrition Control/State Key Laboratory of Swine and Poultry Breeding Industry Guangzhou 510642 China

**Keywords:** roof plate-specific spondin 1 (RSPO1), hair follicle stem cells, hair regeneration, Wnt/β-catenin signaling pathway, mice

## Abstract

Roof plate-specific spondin 1 (R-spondin1, RSPO1) is a Wnt/β-catenin signaling pathway activator that binds with Wnt ligands to stimulate the Wnt/β-catenin signaling pathway, which is key to hair regeneration. However, it is not clear whether recombinant RSPO1 (rRSPO1) affects hair regeneration. Here, we treat C57BL/6 male mice with rRSPO1 and investigate the expression of the Wnt/β-catenin signaling pathway and the activation of hair follicle stem cells in the dorsal skin. The mouse skin color score and hair-covered area are determined to describe hair growth, and the skin samples are subjected to H&E staining, western blot analysis and immunofluorescence staining to evaluate hair follicle development and the expressions of Wnt/β-catenin signaling pathway-related proteins. We find that rRSPO1 activates mouse hair follicle stem cells (mHFSCs) and accelerates hair regeneration. rRSPO1 increases the hair-covered area, the number of hair follicles, and the hair follicle diameter and length. Moreover, rRSPO1 enhances the activity of Wnt/β-catenin signaling pathway-related proteins and the expressions of HFSC markers, as well as mHFSC viability. These results indicate that subcutaneous injection of rRSPO1 can improve hair follicle development by activating the Wnt/β-catenin signaling pathway, thereby promoting hair regeneration. This study demonstrates that rRSPO1 has the potential to treat hair loss by activating the Wnt/β-catenin signaling pathway.

## Introduction

The hair follicle is the basic structure of hair and is not only a sensory organ but also a vector for social interaction in human society. Hair loss disorders, such as androgenetic alopecia and alopecia areatas, are some of the most common types of hair loss in both men and women, affecting approximately 0.2%–2% of the world’s population [ 1, 2] . Hair loss not only damages the patient’s skin function but also affects psychological health. Currently, pharmacological treatments for hair growth disorders often have adverse effects, such as skin disorders and sexual dysfunction [ 3, 4] . Therefore, safe, effective and economical alternative strategies for hair loss treatment have received increasing attention.

The hair follicle is a skin appendage derived from ectoderm, a specific organ of epithelial and mesenchymal interactions. The dermal papilla is required for hair follicle development and promotes proliferation and differentiation [5]. The bulge is a prominent structure of the distal outer root sheath and is a permanent component of the hair follicle that contains hair follicle stem cells (HFSCs) [6]. The hair follicle spontaneously undergoes cycles of growth (anagen), apoptosis (catagen), and quiescence (telogen). The ability of the hair follicle to maintain this cycle is determined by the HFSC pool, the bulge [7]. For hair follicle cycle initiation, the exchange of signals between the dermal papilla and HFSCs is essential. Multiple signaling pathways are involved in this process, such as the wingless (Wnt), bone morphogenetic protein (BMP), epidermal growth factor (EGF), and Sonic Hedgehog (SHH) pathways, among which the Wnt signaling pathway is crucial for entering the anagen phase. The canonical Wnt/β-catenin signaling pathway is required for the proliferation and differentiation of HFSCs, which play a crucial role in hair follicle development [8]. Several hair regenerative disorders are caused by the inability of the hair follicle to reenter the anagen phase of the hair cycle [9]. Activation of the Wnt/β-catenin signaling pathway contributes to the transition from telogen to anagen.

Roof plate-specific spondin 1 (R-spondin1, RSPO1), a member of the R-spondin family of secreted proteins, acts as an agonist of the Wnt/β-catenin signaling pathway and plays essential roles in many processes, including cell proliferation and metabolic homeostasis [10]. The R-spondin family is highly conserved throughout vertebrate evolution and binds with stem cell-specific leucine-rich repeat-containing G-protein-coupled receptors (LGRs). Mechanistically, R-spondin strongly increases the binding of Wnt ligands to initiate Wnt/β-catenin signaling via LGR receptors, which promotes the nuclear accumulation of β-catenin [11]. Once in the nucleus, β-catenin acts as a coactivator for transcription factors to work on Wnt target genes. Previous studies have revealed that RSPO1 is crucial for tissue development and adult tissue homeostasis in tissues such as skin, intestine and muscle [ 12, 13] . Currently, recombinant proteins are used as one method to treat various diseases. Recombinant proteins, such as recombinant human cyclophilin A, recombinant murine IL-36α and recombinant human fibroblast growth factor, have the potential to regulate hair growth [ 14– 16] . We constructed the recombinant vector RSPO1-PET-32α and transformed it into the *Escherichia* *coli* Rosetta strain to express recombinant RSPO1 (rRSPO1) protein. rRSPO1 has approximately 83.92% and 84.48% base sequence identity and approximately 84.34% and 91.98% amino acid residue sequence identity in mice and humans, respectively. Moreover, the production of rRSPO1 offers the double advantage of higher yields and lower price. Therefore, we hypothesized that rRSPO1, as a potential hair loss treatment, may transition hair follicles from the telogen to anagen phase in mice.

Here, we assessed whether RSPO1 promotes hair regeneration in a mouse model and developed a novel recombinant protein, rRSPO1. We demonstrated that rRSPO1 induces the activation of the Wnt/β-catenin signaling pathway. Activation of the Wnt/β-catenin signaling pathway may be associated with mouse HFSC (mHFSC) proliferation and differentiation. Together, our study provides support for the effects of RSPO1 on mouse hair regeneration and a recombinant protein to treat hair loss.

## Materials and Methods

### Production and purification of rRSPO1

As described previously by our laboratory [17], rRSPO1 was expressed in the *Escherichia* *coli* Rosetta strain, separated rapidly by Ni-chelating affinity chromatography (Sangon, Shanghai, China) and further purified. The protein was lyophilized and then reconstituted in sterile phosphate-buffered saline (PBS) prior to use.

### Shaving and depilation of mice

Male C57BL/6 mice were purchased from Guangdong Medical Laboratory Animal Center (Foshan, China). At 8 weeks of age, when the dorsal skin was at the telogen stage, mice were anaesthetized by intraperitoneal administration of sodium pentobarbital and then shaved and depilated. The animal experiment procedures in this study were approved by the Animal Ethics Committee of South China Agricultural University (SCAU#0158; Guangzhou, China) and guided by the Care and Use of Laboratory Animals of South China Agricultural University.

### Subcutaneous injection of rRSPO1

A total of 9 mice were selected for the screening experiments and were randomly divided into three treatment groups with 3 mice per group. The different groups were injected with PBS, rRSPO1 (150 μg/kg, dissolved in PBS) or rRSPO1 (300 μg/kg, dissolved in PBS). Each mouse received a multiple-point subcutaneous injection for 7 consecutive days at the center of the back after depilation.

A total of 16 mice were selected for the formal trial and were randomly divided into two treatment groups with 8 mice per group. The day after depilation, the mice were injected subcutaneously at multiple points with PBS alone (control group) or rRSPO1 (150 μg/kg, dissolved in PBS) for 7 consecutive days. The mice were maintained under conventional conditions at 22–25°C during the hair depilation and photographing procedures. The mouse body weight was measured once a day after hair depilation. The mice were sacrificed using cervical neck dislocation before dorsal skin collection, and the skin samples were stored at –80°C or in 4% paraformaldehyde until further use.

### Isolation and culture of mHFSCs

mHFSCs were isolated and cultured following a previously published protocol [ 18, 19] . Briefly, hair follicles were microdissected from a skin sample under an MZ62 dissecting microscope (Mshot, Guangzhou, China). The hair follicles were washed with PBS and digested with 0.2% collagenase I (Solarbio, Beijing, China) for 30 min at 37°C. Next, the digested samples were centrifuged and seeded in 6-well plates. The cells were cultured in complete medium consisting of 80% DMEM/F12 (MIKX, Shenzhen, China) and 20% fetal bovine serum (MIKX) at 37°C with 5% CO _2_. The culture medium was refreshed every other day. For subsequent experiments, the cells were digested with trypsin when the culture reached a confluency of 80% and subcultured in 6-well plates.

### Cell viability assay

MTT assay was used to detect cell viability. mHFSCs were seeded at 3×10 ^3^ cells/well in 96-well plates (Jet Bio-Filtration, Guangzhou, China) and treated with rRSPO1 or iCRT3 (an inhibitor for β-catenin-responsive transcription, HY-103705; MedChemExpress, Shanghai, China) for 24, 48 and 72 h.Then, 20 μL of MTT solution (Sigma-Aldrich, St Louis, USA) was added to the cells and incubated for 4 h. The crystals were dissolved in 150 μL of DMSO (Sigma-Aldrich) for 30 min and the absorbances were detected with a microplate reader (Bio-Rad, Hercules, USA) at a wavelength of 490 nm.

### Cell proliferation assay

Cell Counting Kit-8 assay was performed to determine cell proliferation activity. After treatment, 10 μL of CCK-8 solution (Sigma-Aldrich) was added to each well and incubated for 4 h. The absorbances were detected with the microplate reader at a wavelength of 450 nm.

### Hematoxylin and eosin (H&E) staining

The dorsal skin samples were fixed with 4% paraformaldehyde overnight and then washed with PBS, dehydrated with alcohol, and embedded in paraffin blocks. Sections of 5 μm were cut with a microtome (Microm-HM340E; Thermo Fisher Scientific, Waltham, USA) and stained with H&E. Then, images were captured under a microscope (Ti2-U; Nikon, Tokyo, Japan), and the number of hair follicles, hair follicle diameter and hair follicle length were measured using ImageJ software ( https://imagej.nih.gov).

### Western blot analysis

Treated mice were sacrificed on days 7 and 14, and dorsal skin samples were collected. The dorsal skin samples were frozen in liquid nitrogen and ground to powder, and the proteins were extracted using radioimmunoprecipitation assay (RIPA) lysis buffer (BioTeke, Beijing, China) containing 0.1% phenylmethylsulfonyl fluoride (PMSF; Sigma-Aldrich). The protein concentrations were quantified using a BCA protein assay kit (Thermo Fisher Scientific) and mixed with 5× SDS-PAGE loading buffer (GenStar, Beijing, China). The proteins and marker (M222; GenStar) were separated by 10% SDS-PAGE (Beyotime Biotechnology, Shanghai, China) and transferred to polyvinylidene fluoride (PVDF) membranes (Millipore, Darmstadt, Germany), which were washed with TBST and blocked in 5% skim milk. Then, the membranes were incubated with primary antibodies and the corresponding secondary antibodies. Finally, after the membrane was immunoreacted with an electrochemiluminescence reagent (P0018FS; Beyotime Biotechnology) and detected with a chemiluminescence system (Protein Simple, Santa Clara, USA). The band densities were analysed with ImageJ software and relative expression levels are shown as fold changes to the control group (arbitrary unit). The primary antibodies used were as follows: LRP6 (sc-25317; Santa Cruz, Dallas, USA), LGR5 (#A503316; Origene, Rockville, USA), active β-catenin (19807; Cell Signaling Technology, Danvers, USA), β-catenin (201328; Zen BioScience, Chengdu, China), TCF4 (ab130014; Abcam, Cambridge, USA), Cyclin D1 (sc-753; Santa Cruz), PCNA (200947-6B12; Zen BioScience), CD34 (14034182; Thermo Scientific, Fremont, USA), cytokeratin 14 (ab181595; Abcam), SOX9 (380995; Zen BioScience), and β-actin (600149; Zen BioScience). HRP-conjugated anti-rabbit IgG (511203; Zen BioScience) and anti-mouse IgG (511103; Zen BioScience) were used as the secondary antibodies.

### Immunofluorescence staining

The dorsal skin sections and mHFSCs were permeabilized with 0.1% Triton X-100 for 15 min and blocked in 5% bovine serum albumin (BSA) for 120 min. The samples were incubated with primary antibodies overnight at 4°C. Secondary staining was performed by incubation with a Cy3 or FITC-conjugated secondary antibody (Jackson Laboratory, Jackson, USA) at room temperature. Finally, the nuclei were stained with 4′,6-diamidino-2-phenylindole (DAPI; Sigma-Aldrich), and images of tissue sections were obtained using a fluorescence microscope (Ti-2 U; Nikon, Tokyo, Japan). Relative expression levels are shown as fold changes to the control group (arbitrary unit).

### Statistical analysis

Results were analysed using SPSS software (version 19.0; SPSS, Chicago, USA) and data are presented as the mean±SEM. The comparisons between two groups were performed by *t* test and among three groups by one-way analysis of variance (ANOVA) and Tukey’s test. Differences were considered statistically significant at *P*<0.05.

## Results

### rRSPO1 accelerates the hair follicle cycle transition

**Figure 1:**
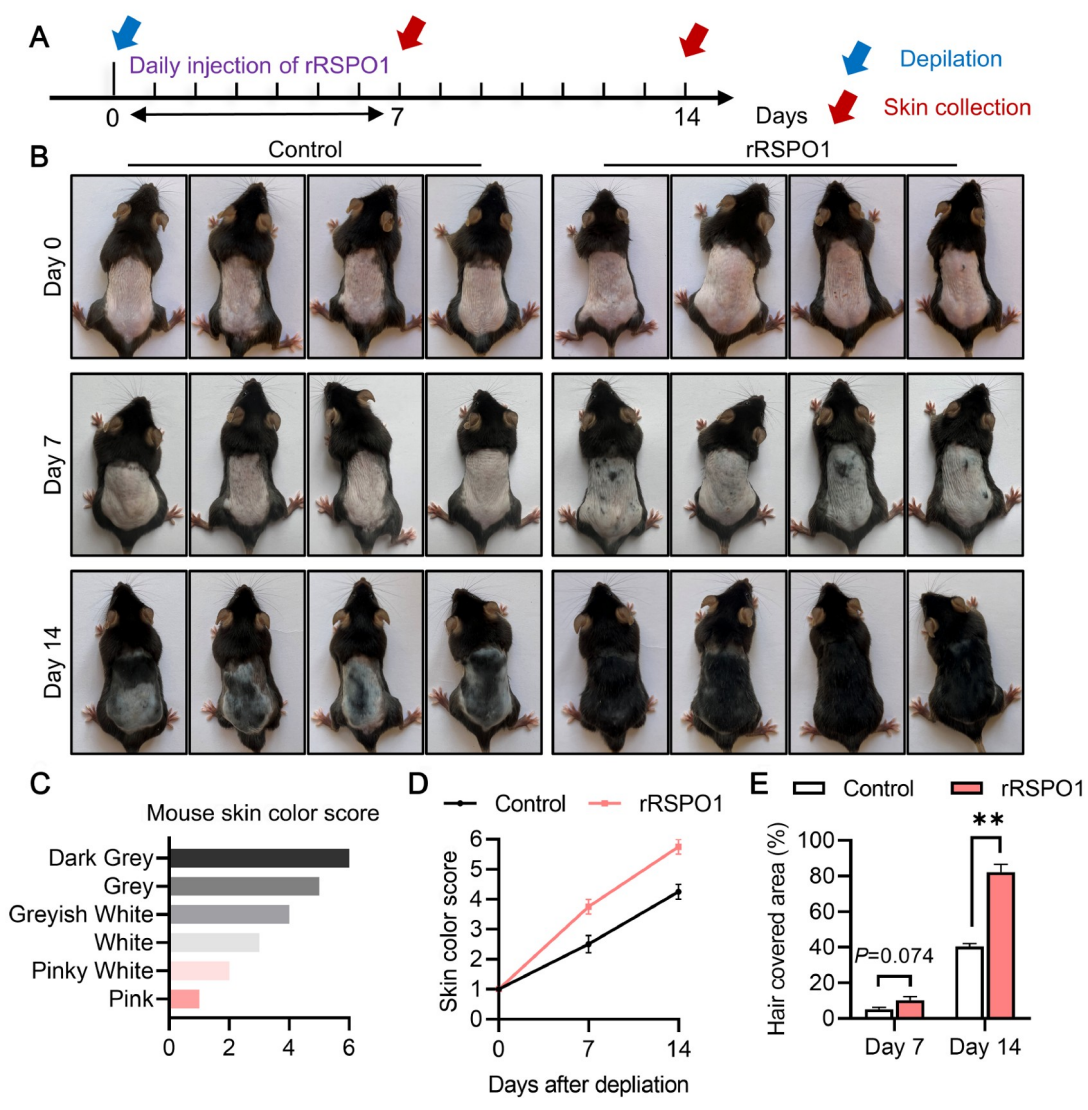
Figure 1 rRSPO1 induces hair follicle growth in C57BL/6 mice (A) Mouse treatment schedule. Male 8-week-old C57BL/6 mice ( n=8 per group) were treated with rRSPO1 beginning on the day after depilation for 7 consecutive days. Blue arrow, the day after depilation; red arrows, start of rRSPO1 treatment. (B) Dorsal hair regeneration of C57BL/6 mice treated with PBS (control) or rRSPO1 (150 μg/kg). (C) Mouse skin color score index. (D) Quantification of the skin color score in the mice depicted in (B) based on the mouse skin color score in (A). (E) Hair-covered area. Data are presented as the mean±SEM. * P<0.05, ** P<0.01.

To test whether rRSPO1 is involved in hair regeneration in mice, on the basis of screening results ( Supplementary Figure S1), we treated mice with rRSPO1 and monitored the hair morphological changes ( Figure 1A). The results showed that rRSPO1 improved hair growth compared to the control at Day 7 and Day 14 ( Figure 1B). The degree of hair growth was scored using skin color as an indicator, and the results showed that hair growth was significantly enhanced in the rRSPO1 group compared to that in the control group ( Figure 1C,D). We estimated the hair-covered area in the dorsal depilation area of the mice and found that the fastest hair-growing phase was from Day 7 to Day 14 in both the control and rRSPO1 groups ( Figure 1E). Compared with the control group, the rRSPO1 group had an increasing trend in hair growth on Day 7 ( *P*=0.074; Figure 1E) and had significantly increased hair growth on Day 14 ( *P*<0.01; Figure 1E). These results revealed that rRSPO1 can efficiently accelerate hair regeneration.

### rRSPO1 improves hair follicle structure

**Figure 2:**
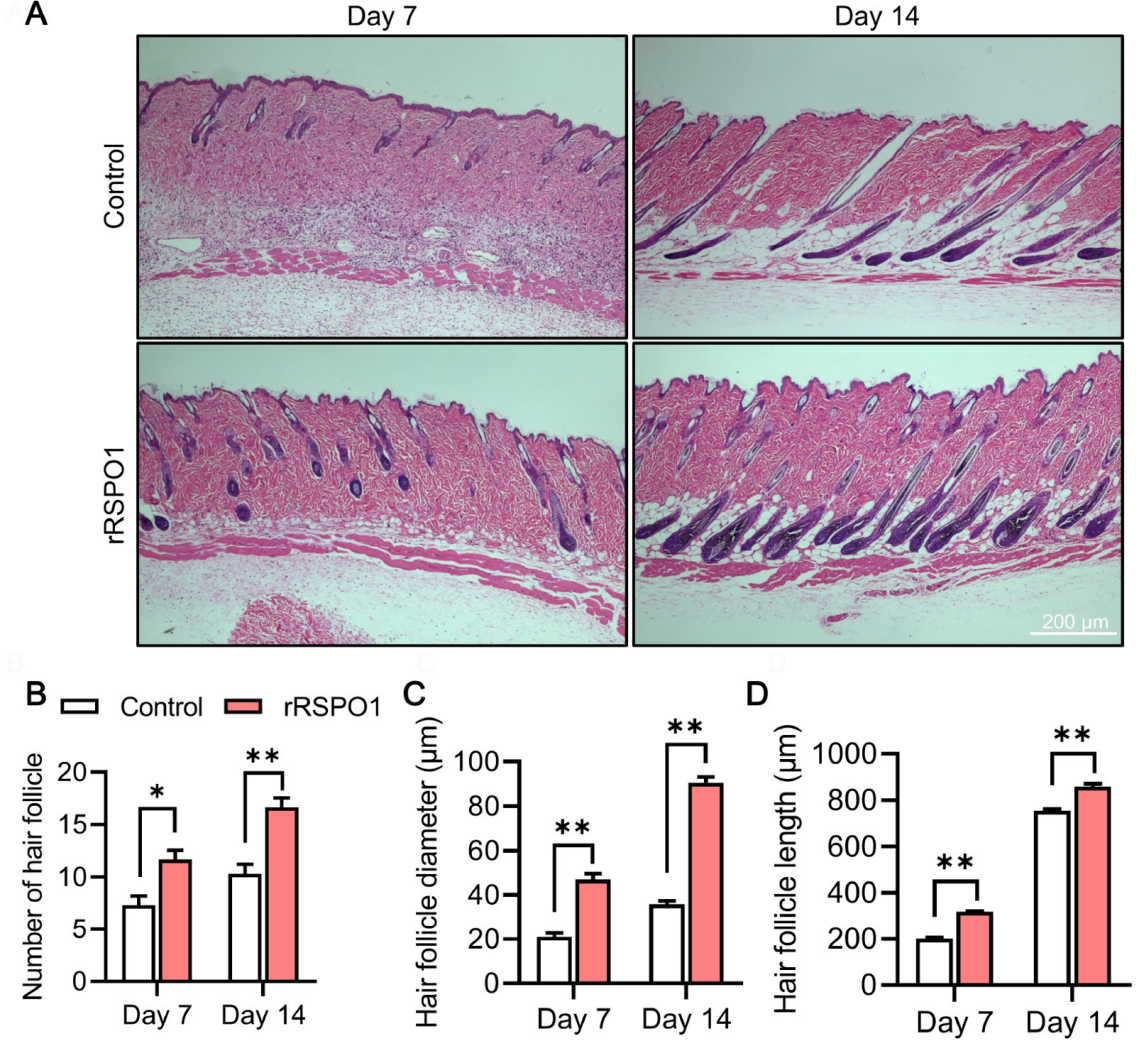
Figure 2 rRSPO1 accelerates hair regrowth and alters the number of hair follicles, hair follicle diameter and hair follicle length (A) H&E staining images (40×) of the dorsal skin of mice treated with PBS (control) or rRSPO1 (150 μg/kg) on days 7 and 14 after depilation. (B) Numbers of hair follicles on days 7 and 14. (C) Hair follicle diameter on days 7 and 14. (D) Hair follicle length on days 7 and 14. Data are presented as the mean±SEM. * P<0.05, ** P<0.01.

To analyse the effect of rRSPO1 on hair follicle development in mice, H&E staining was used to observe the histological changes in the depilated mice ( Figure 2A). H&E staining confirmed that the number of hair follicles was significantly increased in the rRSPO1-treated mice on Day 7 ( *P*<0.05) and Day 14 compared to that of the control mice ( *P*<0.01; Figure 2B). In addition, the rRSPO1-treated mice had a significantly increased diameter and length of hair follicles on Day 7 ( *P*<0.01) and Day 14 ( *P*<0.01; Figure 2C,D). These data indicated that rRSPO1 is highly effective in improving hair regrowth.

### rRSPO1 promotes the telogen-to-anagen transition by enhancing the Wnt/β-catenin signaling pathway

**Figure 3:**
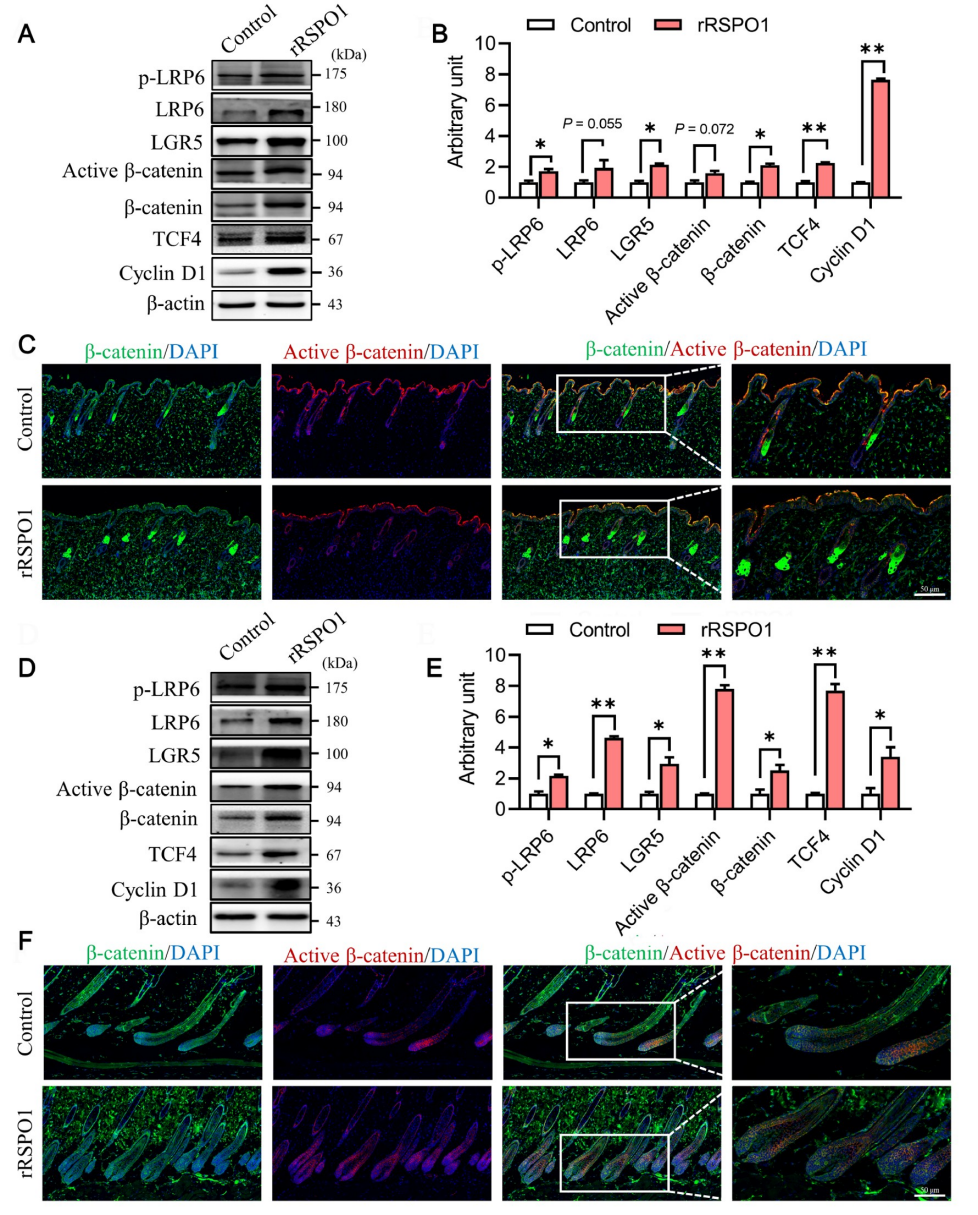
Figure 3 Effect of rRSPO1 on Wnt/β-catenin in mouse dorsal skin at day 7 and day 14 after depilation (A,B) Western blot analysis of the expressions of Wnt/β-catenin-related proteins at day 7 after hair depilation. (C) Immunofluorescence analysis of the expressions of β-catenin and active β-catenin at day 7 after depilation. (D,E) Western blot analysis of the expressions of Wnt/β-catenin-related proteins at day 14 after depilation. (F) Immunofluorescence analysis of the expressions of β-catenin and active β-catenin at day 14 after depilation. Scale bar: 50 μm. Data are presented as the mean±SEM. * P<0.05, ** P<0.01.

RSPO1 is an activator of Wnt signaling. To test this characteristic, we first examined the expressions of members of the Wnt/β-catenin signaling pathway by western blot analysis. As shown in Figure 3A, we found that the Wnt/β-catenin signaling pathway was enhanced after rRSPO1 treatment. The active β-catenin level trended upwards ( *P*=0.072), and the levels of LRP6, LGR5, β-catenin and TCF4 were significantly increased ( *P*<0.05; Figure 3B). In particular, Cyclin D1 expression was significantly increased ( *P*<0.01; Figure 3B). We performed immunofluorescence staining to examine the expressions of active β-catenin and β-catenin in hair follicles. Compared with that of the control, increased nuclear accumulation of β-catenin was observed in the bulge. The fluorescence signals of active β-catenin and β-catenin in hair follicles were increased ( Figure 3C). Consistently, the results suggest that rRSPO1 activates the Wnt/β-catenin signaling pathway. Next, we further studied whether rRSPO1 promotes the expression of the Wnt/β-catenin signaling pathway during the anagen phase. Consistent with the results on Day 7, western blot analysis and immunofluorescence staining results showed that the expression of the Wnt/β-catenin signaling pathway was significantly increased by rRSPO1. The levels of LGR5, β-catenin and Cyclin D1 were increased ( *P*<0.05), and the levels of LRP6, active β-catenin and TCF4 were significantly increased ( *P*<0.01; Figure 3D,E). The fluorescence signals of active β-catenin and β-catenin in hair follicles were increased ( Figure 3F). These results suggested that rRSPO1 potentiates the Wnt/β-catenin signaling pathway.

### Induction of HFSC proliferation and differentiation by rRSPO1

**Figure 4:**
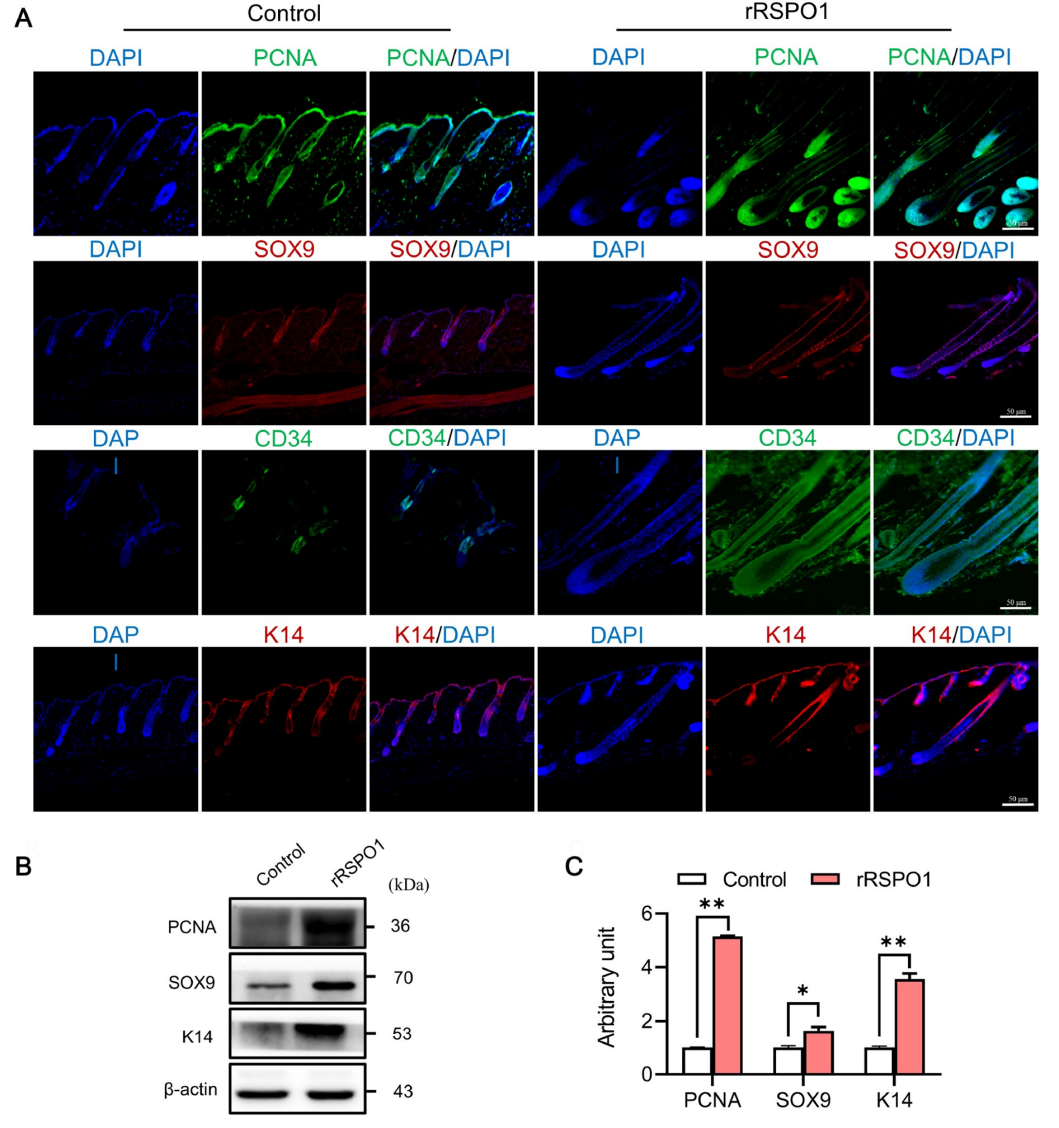
Figure 4 Effect of rRSPO1 on PCNA, CD34, K14 and SOX9 in mouse dorsal skin at day 14 after depilation (A) Immunofluorescence analysis of the expressions of PCNA, CD34, K14 and SOX9 at day 14 after depilation. Scale bar: 50 μm. (B,C) Western blot analysis of the expressions of PCNA, K14 and SOX9 at day 14 after depilation. Data are presented as the mean±SEM. * P<0.05, ** P<0.01.

To investigate the role of rRSPO1 in the telogen-to-anagen transition, we examined proliferation and differentiation factors in dorsal skin. The rRSPO1-treated and control hair follicles were morphologically similar. Thus, we examined the expressions of proliferation and differentiation factors in hair follicles ( Figure 4A). The number of proliferative cells was increased in the rRSPO1 matrix, suggesting that rRSPO1 promotes cell proliferation. Hair regeneration arises from HFSCs. We analysed the expressions of the HFSC markers CD34 and K14. The bulge was positive for CD34 and K14 in controls. In contrast, the rRSPO1-treated hair follicles were positive for CD34 and K14 in the outer root sheath of the isthmus and the upper suprabulbar region. To further investigate the regulation of keratinocyte differentiation by rRSPO1, we first examined transcription factors that are important for keratinocyte differentiation. Sox9 is a transcription factor that promotes the differentiation of HFSCs into the outer root sheath, which is essential for outer root sheath differentiation and the formation of the hair stem cell compartment. Consistent with the above expressions of HFSC markers, Sox9 positivity was localized in the bulge region in control hair follicles, whereas in the rRSPO1-treated hair follicles, Sox9 positivity was most intense in the inferior portion of the hair follicle and diminished from the hair bulb toward the infundibulum. Subsequently, we examined the expression levels of the above proteins by western blot analysis. Western blot analysis results of PCNA, K14, and Sox9 in the rRSPO1-treated hair follicles suggested that proliferation and differentiation factors were re-expressed in hair follicles ( Figure 4B,C). These data suggested that rRSPO1 plays an important role in matrix cell formation, proliferation, and differentiation.

### rRSPO1 promotes mHFSC viability by targeting Wnt/β-catenin signaling

**Figure 5:**
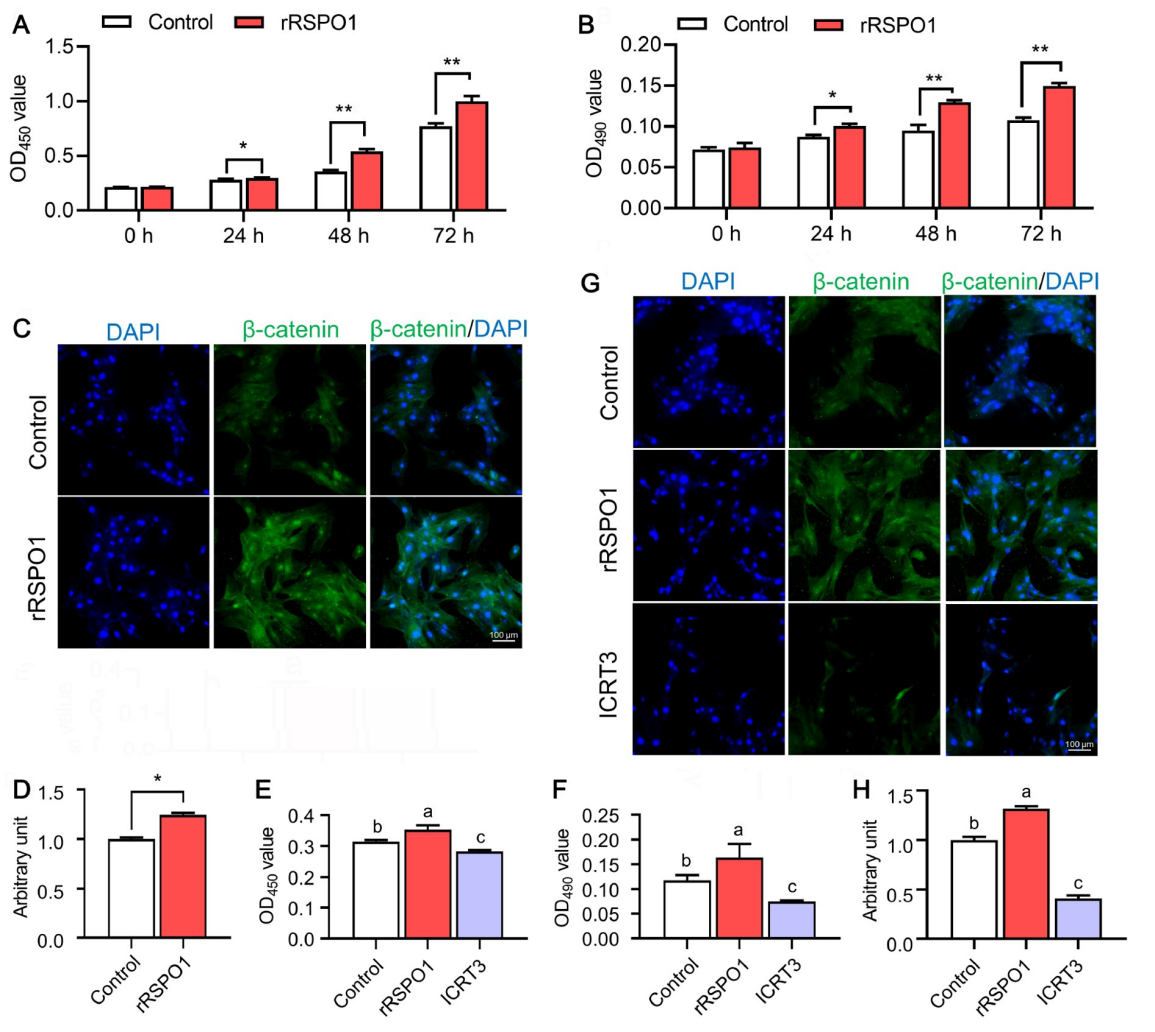
Figure 5 rRSPO1 promotes mHFSC viability by targeting Wnt/β-catenin signalling (A,B) mHFSCs were treated with 0 or 300 ng/mL rRSPO1. The cell viability in the two groups at 24, 48 and 72 h was measured by CCK-8 and MTT assays, n=8 wells per group. (C,D) Immunofluorescence analysis of the expression of β-catenin after 24 h of treatment with 0 or 300 ng/mL rRSPO1. Scale bar: 100 μm. (E,F) mHFSCs were treated with 0 or 300 ng/mL rRSPO1 or 300 ng/mL rRSPO1+5 nM iCRT3. The cell viability in the two groups at 24 h was measured by CCK-8 and MTT assays, n=8 wells per group. (G,H) Immunofluorescence analysis of the expression of β-catenin after 24 h of treatment with 0 or 300 ng/mL rRSPO1 or 300 ng/mL rRSPO1+5 nM iCRT3. Data are presented as the mean±SEM. * P<0.05, ** P<0.01; for “a–c”, the different letters indicate significant difference ( P<0.05).

To further investigate the effect of rRSPO1 on the viability of mHFSCs, we isolated and cultured mHFSCs from mice. Base on our concentration screening results, we chose the optimal concentration of 300 ng/mL for further analysis ( Supplementary Figure S2). mHFSCs were cultured in DMEM/F12 with 300 ng/mL rRSPO1 for 24, 48 and 72 h. The MTT assay and CCK-8 assay results showed that treatment with 300 ng/mL rRSPO1 for 24, 48 and 72 h significantly affected the viability and proliferation of mHFSCs ( Figure 5A,B). The optimal concentration of rRSPO1 to promote mHFSC viability was 300 ng/mL rRSPO1 for 24 h. mHFSCs were further analysed by examining Wnt/β-catenin signaling using immunofluorescence staining. Compared with the control, treatment with 300 ng/mL rRSPO1 significantly increased the level of β-catenin ( *P*<0.05; Figure 5C,D). Next, to evaluate the effect of Wnt/β-catenin signaling on cell viability of mHFSCs, we used an inhibitor of both Wnt and β-catenin-responsive transcription, iCRT3, to decrease signaling through Wnt/β-catenin signaling ( Supplementary Figure S2). As shown in Figure 5E,F, 5 nM iCRT3 significantly reduced the viability of mHFSCs ( *P*<0.01). Apparently, iCRT3 inhibits the effect of rRSPO1 and reduces Wnt/β-catenin signaling activity ( Figure 5G,H). These data inciated that ICRT3 reduces the proliferation of mHFSCs by inhibiting Wnt/β-catenin signalling.

## Discussion

The hair follicle cycle consists of three phases: anagen, catagen and telogen. The differentiated melanocyte stem cell progeny synthesizes and transfers pigments to the events of the epithelial cells at the anagen phase, which can darken the skin. In contrast to the anagen phase, differentiated melanocyte apoptosis occurs in the telogen phase, which turns the skin pink [20]. Thus, mouse skin color can be used as direct evidence to judge the hair follicle cycle. In our study, rRSPO1 was injected into mice at 8 weeks of age because mouse hair follicles are in the telogen phase and the skin is pink at this time [21]. We observed that the skin turned black after rRSPO1 treatment, which demonstrates that the hair follicles entered the anagen phase.

Hair follicle formation and maintenance are regulated by multiple signaling pathways in strict temporal and spatial patterns [22]. Wnt signaling pathways seem to be dominant in epidermal-dermal communication [23]. In particular, the canonical Wnt/β-catenin signaling pathway is responsible for the hair follicle fate switch [24]. The Wnt/β-catenin signaling pathway gradually decreases to a minimum in the telogen phase and then continues to be highly active until the end of the anagen phase. This suggests that during HFSC development, the Wnt/β-catenin signaling pathway promotes the telogen-to-anagen transition of the hair follicle cycle [25]. In the presence of Wnt ligand, Wnt ligand forms a ternary complex with LRP6 and Frizzled (FZD) and clusters together with the scaffold protein Dishevelled to form a signalosome for endocytosis in the cytoplasm [26]. The signalosome further forms multivesicular bodies where LRP6 is sequestered together with the destruction complex and phosphorylated [27]. Phosphorylation of β-catenin is prevented, and β-catenin escapes its degradative fate to transfer Wnt target genes. However, Wnt/β-catenin signaling pathway activity is reduced due to ubiquitination of the ternary complex induced by cell-surface transmembrane E3 ubiquitin ligases [28]. RSPO1 binds to LGRs and E3 ubiquitin ligases, triggering the autoubiquitination of E3 ubiquitin ligases, which amplifies Wnt/β-catenin signaling pathway activity by increasing the availability of the ternary complex [29].

Our results showed that the expression levels of LRP6, LGR5, active β-catenin, β-catenin and TCF4 were significantly higher in the rRSPO1 group than in the control group. Moreover, the expression level of the target protein Cyclin D1 was increased in the rRSPO1 group. This finding suggests that RSPO1 can activate the Wnt/β-catenin signaling pathway and further upregulate the expressions of Wnt target genes. Moreover, we found that DKK1, a Wnt/β-catenin signaling pathway antagonist, is induced by dihydrotestosterone from balding dermal papilla cells, and high expression of DKK1 in mice retards hair follicle cycle transformation [30]. Binnerts *et al*. [31] found that RSPO1 competes with DKK1 for binding to LRP6, which interferes with DKK1-mediated internalization to increase LRP6 levels on the cell surface. Our results are consistent with the findings of Binnerts *et al*. and indicate that increased LRP6 expression is important in promoting hair growth. This implies that the promotion of hair growth by RSPO1 may be associated with interference with DKK1-mediated internalization. Weber *et al*. [32] reported that the expression of RSPO1 was significantly upregulated in the human fetal dermal papilla. The increased expression of RSPO1 in early embryonic development indicates that it is involved in the activation of HFSCs and plays a role in the early stages of hair growth [33]. The adult HFSC pool has an active Wnt/β‐catenin signaling pathway, which is highly prone to differentiation during the hair follicle cycle [34]. Based on our findings, we hypothesized that rRSPO1-mediated Wnt/β‐catenin signaling pathway activation may induce the expression of PCNA and the differentiation of stem cells with CD34, K14 and SOX9 positivity. We suggest that rRSPO1 promotes hair follicle growth by activating the Wnt/β-catenin signaling pathway in HFSCs.

Wnt/β-catenin signaling pathway activators show promise as novel drug candidates. The inability of the hair follicle cycle to transition from the telogen phase to the anagen phase is one of the reasons why hair regeneration is hindered [9]. Recently, several studies have revealed that targeting Wnt/β-catenin can promote the activation or differentiation of HFSCs to induce hair growth. For example, murine skin topically treated with KY19382, a newly synthesized analog of indirubin‐3′‐monoxime which can increase the level of β-catenin in the keratin 15‐positive bulge, showed more robust hair growth [35]. The molecular chaperone cyclophilin A promotes cell proliferation and differentiation through the Wnt/β-catenin signaling pathway and stimulates hair follicle and dermal papilla cells [14]. Therefore, RSPO1 has potential for hair regeneration therapy.

In summary, this study suggests that rRSPO1 could directly target the Wnt/β-catenin signaling pathway in HFSCs to induce HFSC proliferation and differentiation to promote hair regeneration, which may be beneficial for the treatment of hair loss in the future.

## Supplementary Data

Supplementary data is available at *Acta Biochimica et Biphysica Sinica* online.

## Supporting information

476FigS1-S2
