# Supplementary material for: Novel recombinant R-spondin1 promotes hair regeneration by targeting the Wnt/β-catenin signaling pathway: Recombinant R-spondin1 promotes hair regeneration
Source: Acta Biochim Biophys Sin (Shanghai). 2023 Jul 20;55(8):1213–21. doi: 10.3724/abbs.2023112 (PMC10448039; doi:10.3724/abbs.2023112)
Supplement: 476FigS1-S2 [file 476FigS1-S2.pdf]

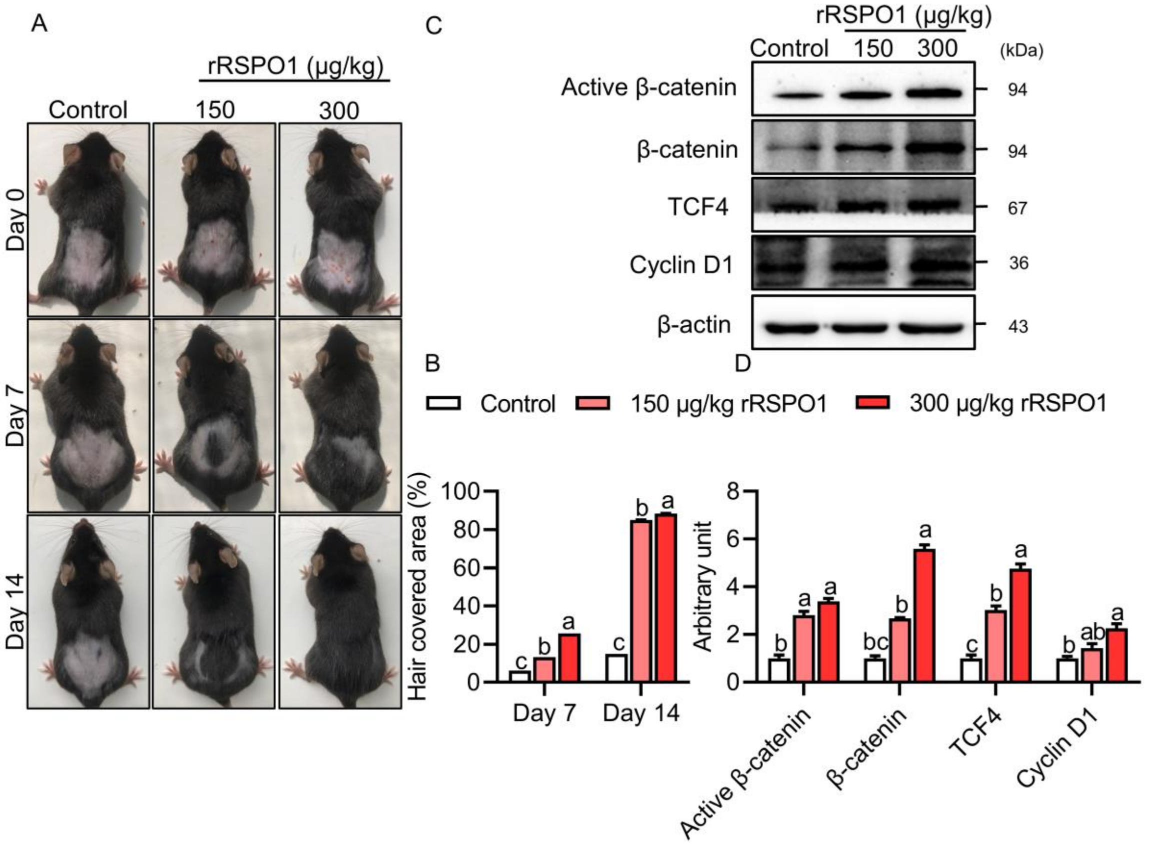

Supplementary Figure S1. rRSOPO1 concentration screening *in vivo* assay (A) Dorsal hair regeneration of C57BL/6 mice treated with PBS (control), 150 μg/kg rRSPO1 or 300 μg/kg rRSPO1 (n = 3 per group). (B) Hair-covered area. (C, D) Western blotting detection of the expression of Wnt/β-catenin-related proteins at Day 14 after depilation. Data are represented as the mean ± SEM. *P< 0.05, **P<0.01.

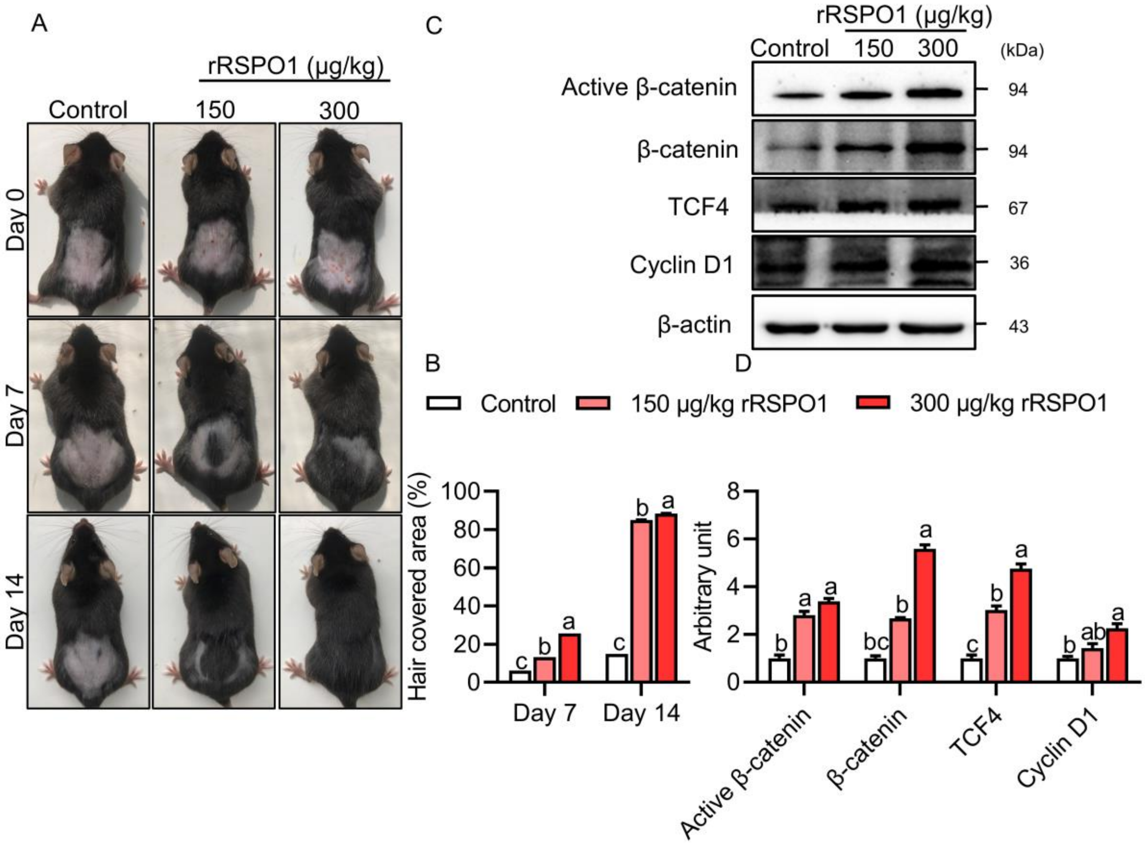
A
rRSPO1 (μg/kg)
Control
150
300
Day 0
Day 7
Day 14
B
Hair covered area (%)
Day 7
Day 14
C
rRSPO1 (μg/kg)
Control 150 300
(kDa)
Active β-catenin 94
β-catenin 94
TCF4 67
Cyclin D1 36
β-actin 43
D
Control
150 μg/kg rRSPO1
300 μg/kg rRSPO1
Arbitrary unit
Active β-catenin
β-catenin
TCF4
Cyclin D1

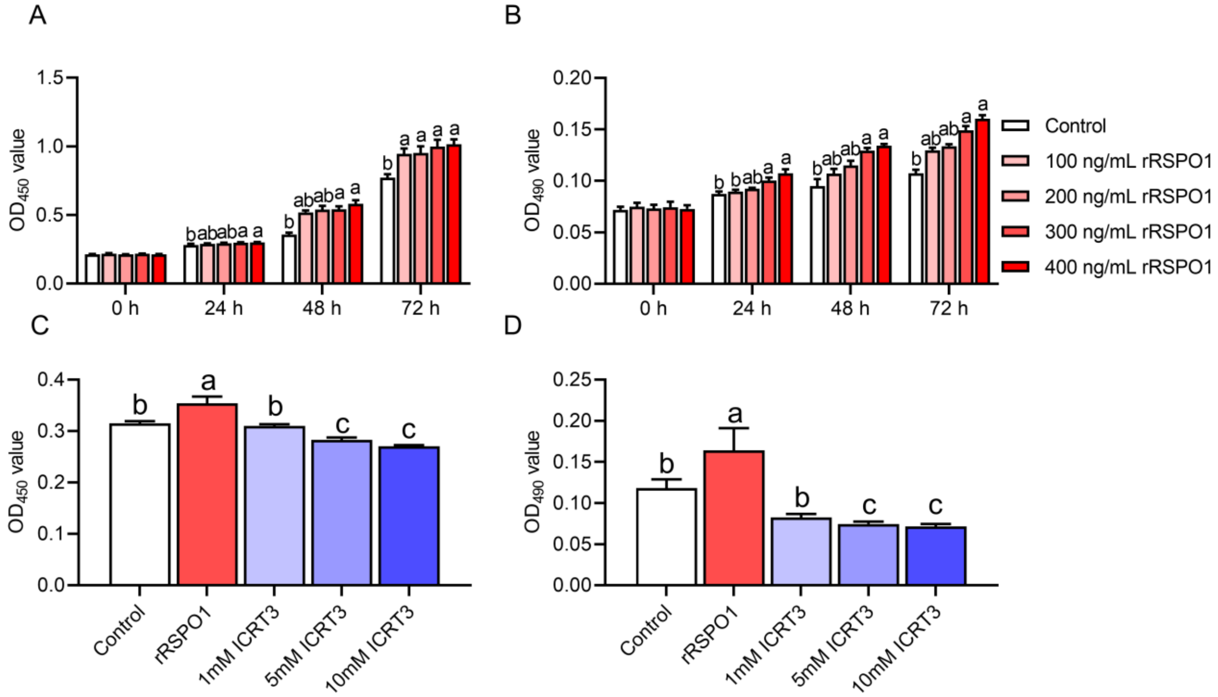
A
OD_{450} value
0 h
24 h
48 h
72 h
B
OD_{490} value
Control
100 ng/mL rRSPO1
200 ng/mL rRSPO1
300 ng/mL rRSPO1
400 ng/mL rRSPO1
C
OD_{450} value
Control
rRSPO1
1mM ICRT3
5mM ICRT3
10mM ICRT3
D
OD_{490} value
Control
rRSPO1
1mM ICRT3
5mM ICRT3
10mM ICRT3

Supplementary Figure S2. rRSOPO1 concentration screening *in vitro* (A, B). mHFSCs were treated with 0, 100, 200, 300 and 400 ng/mL rRSPO1. The cell viability in the two groups at 24, 48 and 72 h was measured by CCK-8 and MTT assays, $n = 8$ wells per group. (C, D) mHFSCs were treated with 0 or 300 ng/mL rRSPO1, 300 ng/mL rRSPO1 + 1 nM iCRT3, 300 ng/mL rRSPO1 + 5 nM iCRT3 and 300 ng/mL rRSPO1 + 10 nM iCRT3. The cell viability in the two groups at 24 h was measured by CCK-8 and MTT assays, $n = 8$ wells per group. Data are represented as the mean ± SEM. "a-c" indicates $P < 0.05$.
